# Impact of a health education intervention on knowledge, attitudes, and use of visibility materials among commercial motorcycle riders: a quasi-experimental study in Cameroon

**DOI:** 10.21203/rs.3.rs-9163081/v1

**Published:** 2026-03-27

**Authors:** Chrisantus Eweh Ukah, Nicholas Tendongfor, Alan Hubbard, Elvis Asangbeng Tanue, Rasheedat Oke, Nahyeni Bassah, Larissa Kumenyuy Yunika, Claudia Ngeha Ngu, S. Ariane Christie, Dickson Shey Nsagha, Alain Chichom-Mefire, Catherine Juillard

**Affiliations:** University of Buea; University of Buea; University of California, Berkeley; University of Buea; University of California, Los Angeles; University of Buea; University of Buea; University of Buea; University of California, Los Angeles; University of Buea; University of Buea; University of California, Los Angeles

**Keywords:** Road traffic injuries, motorcycle safety, visibility materials, health education, quasi-experimental study, Cameroon

## Abstract

**Background:**

Commercial motorcycle riders are among the most vulnerable road users in low- and middle-income countries, with poor visibility contributing significantly to road traffic crashes, particularly under low-light conditions. Visibility materials such as reflective clothing and functional motorcycle lighting systems are effective, low-cost injury prevention measures; however, their use remains suboptimal. This study evaluated the impact of a theory-driven health education intervention on knowledge, attitudes, and use of visibility materials among commercial motorcycle riders in Cameroon.

**Methods:**

A quasi-experimental, non-randomized controlled before-and-after study was conducted in Limbe (intervention) and Tiko (control) Health Districts. Participants were drawn from a baseline cross-sectional study and followed over an eight-month intervention period. The intervention, guided by the Health Belief Model and developed using the Intervention Mapping framework, combined face-to-face sensitization sessions with mobile phone–based messaging tailored to participants’ literacy levels. Data were collected at baseline and endline using structured questionnaires and direct observation checklists. Knowledge, attitude, and practice scores were treated as continuous variables. Difference-in-differences analysis using generalized estimating equations was employed to estimate the intervention effect, adjusting for socio-demographic factors.

**Results:**

A total of 249 riders were retained at endline (149 intervention, 100 control). In the intervention group, significant improvements were observed in knowledge (mean difference = 1.75, p < 0.001), attitudes (mean difference = 5.60, p < 0.001), and practices (mean difference = 0.36, p = 0.012), while no significant changes were observed in the control group. Difference-in-differences analysis showed that the intervention was associated with significant increases in knowledge (β = 1.68, 95% CI: 1.16–2.20) and attitudes (β = 5.51, 95% CI: 4.22–6.80), but not practices (β = 0.34, 95% CI: −0.11 −0.79). For visibility material use, the intervention was associated with a significant increase in reflective jacket use (AOR = 4.34, 95% CI: 1.88–10.02), while no significant effects were observed for other visibility materials.

**Conclusion:**

A theory-informed health education intervention significantly improved knowledge, attitudes, and reflective jacket use among commercial motorcycle riders. However, improvements in other visibility-related practices were limited, suggesting that educational strategies alone may be insufficient to address structural and resource-related barriers. Multi-level interventions combining education with improved access and enforcement are needed to achieve sustained adoption of visibility materials for injury prevention.

**Trial registration:**

ClinicalTrials.gov Identifier: NCT07087444. Registered on July 28, 2025.

## Background

Road traffic injuries remain a major public health challenge worldwide, causing approximately 1.19 million deaths each year and representing one of the leading causes of death and disability, particularly in low- and middle-income countries[1]. Motorcyclists constitute one of the most vulnerable groups of road users due to the limited physical protection offered by motorcycles and their high exposure to traffic hazards[2–4]. In many urban and peri-urban areas of sub-Saharan Africa, commercial motorcycle transport has emerged as a major source of livelihood and an essential mode of mobility. However, this rapid expansion has been accompanied by a growing burden of motorcycle-related crashes, injuries, and fatalities[5].

A substantial proportion of motorcycle crashes occur under conditions of reduced visibility, including during early morning hours, at night, or in poor weather and lighting conditions. Low conspicuity of motorcycles and their riders have consistently been identified as an important contributor to collisions with other road users[6–9]. Compared with larger vehicles, motorcycles are smaller, less visually salient, and more easily obscured in traffic, increasing the likelihood that drivers fail to detect them in time to avoid a crash.

Visibility materials are therefore an important component of motorcycle safety. These include reflective jackets or vests worn by riders, reflective strips mounted on motorcycles, and functional lighting systems such as headlamps, rear lights, and indicators[10]. Such materials enhance rider conspicuity, increase detection distance for other road users, and provide additional reaction time to prevent collisions. Evidence from multiple settings indicates that the use of reflective and high-visibility materials improves detection of motorcycles in traffic and is associated with a reduced risk of road traffic crashes, particularly in low-light environments[11, 12].

Despite these documented benefits, the uptake and consistent use of visibility materials among commercial motorcycle riders in many low-resource settings remains limited[13]. Several barriers have been reported, including limited awareness of their protective value, misconceptions about their effectiveness, discomfort during use, financial constraints, and weak enforcement of road safety regulations[14]. In addition, behavioural and social factors such as peer influence, risk perception, and prevailing community norms can influence riders’ willingness to adopt safety practices.

In Cameroon, commercial motorcycle transport, commonly referred to as “okada,” plays a central role in daily mobility, particularly in urban and peri-urban areas where formal public transportation systems are limited[5]. In the South-West Region, including the Limbe and Tiko Health Districts, commercial motorcycle riding has expanded rapidly. This growth has been partly driven by economic hardship and the ongoing socio-political crisis, which has disrupted traditional livelihoods and increased reliance on informal transport services. These districts are characterized by dense traffic, mixed road use, variable road infrastructure, and frequent night-time riding, all of which increase the importance of rider conspicuity. However, the use of reflective clothing and other visibility-enhancing materials among riders in these settings remains inconsistent, and evidence on effective strategies to improve their uptake is limited.

Improving the adoption of visibility materials requires more than simply making protective equipment available. Behavioural change is influenced by individuals’ perceptions of risk, beliefs about the severity of injuries, perceived benefits of protective actions, perceived barriers to behaviour change, and confidence in their ability to adopt and maintain safe practices. The Health Belief Model provides a useful theoretical framework for understanding these behavioural determinants. According to this model, engagement in preventive health behaviours is influenced by perceived susceptibility to harm, perceived severity of potential consequences, perceived benefits of protective actions, perceived barriers to action, cues that trigger behaviour, and self-efficacy[15].

In addition to theoretical understanding, the development of effective public health interventions requires systematic planning that considers local context and stakeholder engagement. The Intervention Mapping approach offers a structured framework for translating behavioural theory and empirical evidence into practical intervention strategies[16]. This approach emphasizes identifying behavioural and environmental determinants, developing theory-based change methods, designing culturally appropriate intervention materials, and planning for implementation and evaluation. Intervention Mapping is particularly useful for complex public health challenges such as road traffic injury prevention, where individual behaviours interact with broader social and environmental factors.

Despite growing recognition of the role of rider conspicuity in motorcycle safety, there remains a paucity of intervention studies in sub-Saharan Africa that specifically evaluate strategies to improve the use of visibility materials among commercial motorcycle riders. Most studies conducted in the region have focused primarily on helmet use and other forms of personal protective equipment, with relatively little attention given to visibility-related safety practices.

To address this gap, the present study evaluated the impact of a theory-driven health education intervention designed to improve knowledge, attitudes, and the use of visibility materials among commercial motorcycle riders in the Limbe and Tiko Health Districts of Cameroon. The intervention was informed by the Health Belief Model to guide behavioural targets and messaging and was developed using the Intervention Mapping approach to ensure systematic planning and contextual relevance. By strengthening evidence on behavioural strategies to promote rider conspicuity, this study aims to contribute to locally relevant and scalable road safety interventions for reducing motorcycle-related crashes in high-risk urban settings.

## Materials and Methods

### Study design

A quasi-experimental, non-randomized controlled before-and-after study design was used to evaluate the effectiveness of a health education intervention aimed at improving knowledge, attitudes, and uptake of visibility materials among commercial motorcycle riders. Limbe Health District served as the intervention district, while Tiko Health District served as the comparison district. Allocation of districts to study arms was non-random and was determined based on administrative feasibility and operational considerations. The same cohort of commercial motorcycle riders in both districts was followed from baseline to endline. This design enabled assessment of changes in outcomes over time within each district and estimation of the intervention effect using a difference-in-differences analytical approach. The study protocol was registered with ClinicalTrials.gov (Identifier: NCT07087444; registered on July 28, 2025). The study was registered retrospectively.

### Study setting

The study was conducted in the Limbe and Tiko Health Districts located in Fako Division of the South-West Region of Cameroon. Both districts are characterized by high levels of commercial motorcycle transport activity and serve as major urban and peri-urban transport hubs. The districts have also been affected by the ongoing socio-political crisis in the English-speaking regions of Cameroon, which has disrupted livelihoods and increased reliance on commercial motorcycle transport as a source of income. Road networks in both districts are heavily utilized for commuting and commercial transport, and commercial motorcycle riders constitute a major group of vulnerable road users due to frequent exposure to dense traffic, mixed road use, and night-time riding conditions.

### Study population

The study population consisted of commercial motorcycle riders operating within the Limbe and Tiko Health Districts. Riders were eligible for participation if they were aged 18 years or older, had been operating as commercial motorcycle riders in the study area for at least three months, owned or operated a functional motorcycle, possessed a mobile phone, were able to communicate in English or Pidgin English, and provided written informed consent. Riders who declined to participate were excluded from the study.

### Sampling and recruitment

Participants for the quasi-experimental intervention study were drawn from a previously conducted cross-sectional study that assessed knowledge, attitudes, and use of visibility materials among commercial motorcycle riders in the Limbe and Tiko Health Districts. In that cross-sectional study, a total of 499 riders were recruited, including 300 riders from Limbe Health District and 199 riders from Tiko Health District.

During the cross-sectional survey, participants were informed about a planned follow-up health education intervention aimed at improving the use of visibility materials among commercial motorcycle riders. Riders who expressed willingness to participate in the intervention were asked to provide their contact information, including mobile phone numbers, to facilitate future follow-up. These participants formed the sampling frame for the intervention study.

From this pool of riders, those who had provided consent and contact information were invited to participate in the quasi-experimental intervention study. In the intervention district (Limbe), 183 riders from the original cross-sectional sample were successfully recruited and followed up for participation in the intervention. In the control district (Tiko), 130 riders from the original cross-sectional sample were recruited and followed up during the same period.

Participants were traced using the contact information provided during the cross-sectional survey as well as their motorcycle park affiliations. Only riders who had participated in the baseline cross-sectional survey and who consented to follow-up were eligible for inclusion in the intervention study.

### Sample size considerations

The minimum required sample size was estimated based on the expected improvement in knowledge related to road safety practices following the health education intervention. The calculation assumed a baseline knowledge proportion of 32.6%, a minimum detectable increase of 16%, a confidence level of 95%, and a statistical power of 90%. Allowing for potential loss to follow-up, a minimum of 100 riders per study arm was targeted. In practice, 183 riders were enrolled at baseline in the intervention district and 130 riders in the control district. At endline, 149 riders in the intervention district and 100 riders in the control district were successfully followed up.

### Development of the health education intervention

The health education intervention was developed using a theory-driven and participatory approach informed by the Health Belief Model and the Intervention Mapping framework. The development process was grounded in findings from a formative qualitative needs assessment conducted among commercial motorcycle riders and key stakeholders in the Limbe Health District.

### Formative needs assessment

Prior to designing the intervention, a qualitative needs assessment was conducted to identify the behavioural and contextual determinants influencing the use of visibility materials among commercial motorcycle riders. Four focus group discussions were conducted among commercial motorcycle riders operating in Limbe Health District. Each focus group consisted of between six and eight participants, and discussions were conducted until thematic saturation was achieved. In addition, key informant interviews were conducted with stakeholders involved in road safety and motorcycle transport regulation. These included two experienced commercial motorcycle riders, two motorcycle union leaders, the Divisional Delegate of Transport for Fako Division, a road safety police officer, and a healthcare worker involved in injury prevention. The discussions explored perceptions of road traffic injury risk, knowledge and beliefs about the importance of rider visibility, barriers to the use of reflective materials and functional motorcycle lighting systems, and potential strategies for improving safety practices among riders. Findings from the focus group discussions and key informant interviews were used to identify behavioural determinants and inform the development of intervention messages and delivery strategies.

### Theoretical framework for intervention development

The Health Belief Model guided the selection of behavioural determinants and message content for the intervention. The intervention specifically addressed perceived susceptibility to road traffic injuries, perceived severity of injury outcomes, perceived benefits of using visibility materials, perceived barriers to behaviour change, cues to action, and self-efficacy. The Intervention Mapping framework was used to translate the identified needs and determinants into behavioural change objectives and practical intervention strategies. This process involved identifying priority behaviours related to the use of reflective jackets and maintenance of functional motorcycle lighting systems, specifying performance objectives, selecting theory-based behaviour change methods, and developing culturally appropriate intervention materials.

### Structure and delivery of the intervention

The intervention consisted of a combination of face-to-face sensitization sessions and mobile phone–based educational messaging. Three group sensitization sessions were conducted in the intervention district at the beginning of the intervention, two months after initiation, and two months thereafter. These sessions included interactive discussions, demonstrations of reflective jackets and motorcycle lighting systems, clarification of misconceptions regarding rider visibility, and opportunities for participants to share experiences related to road safety practices.

In addition to the physical sessions, weekly educational messages were delivered throughout the intervention period using mobile phone–based communication. The delivery approach was adapted to participants’ literacy levels, phone types, and communication preferences. Riders with smartphones received weekly WhatsApp text messages, each accompanied by a voice recording of the same message to ensure accessibility for those with limited literacy. Riders who did not use smartphones but were able to read received short message service (SMS) messages, while those who preferred verbal communication received periodic phone calls during which the educational messages were delivered verbally. This multi-channel approach ensured accessibility across varying literacy levels.

### Duration of the intervention

The intervention was implemented over a period of eight months, from September 2024 to April 2025.

### Control group

Commercial motorcycle riders in Tiko Health District served as the control group. Participants in the control district continued to receive routine road safety information and services normally available in the district but did not receive any study-related health education on visibility materials during the intervention period.

### Baseline and endline data collection

Baseline data were collected in both districts prior to implementation of the intervention, and endline data were collected immediately after completion of the intervention. The same structured and pre-tested questionnaire was used during both baseline and endline surveys. The questionnaire was developed specifically for this study based on relevant literature and findings from the formative qualitative phase and was pre-tested and refined prior to use. The questionnaire collected information on socio-demographic characteristics, knowledge of visibility materials, attitudes toward rider visibility, and self-reported use of reflective jackets and motorcycle lighting systems. The full questionnaire is provided as Additional file 1.

In addition to self-reported data, direct observations were conducted at motorcycle parks and drop-off points using a structured checklist to assess the actual use of reflective jackets and the functionality of motorcycle lighting systems during routine riding. Data were collected electronically using Kobo Collect by trained data collectors. Interviews were conducted in English, with Pidgin English used where necessary to ensure comprehension.

### Statistical analysis

All analyses were conducted using SPSS version 26. Descriptive statistics were used to summarize participant characteristics and outcome measures at baseline and endline. Attrition analyses were conducted separately for the intervention and control groups to compare baseline socio-demographic characteristics between participants retained at endline and those lost to follow-up using chi-square tests.

Within-group changes between baseline and endline were examined separately for the intervention and control groups. Paired samples t-tests were used to evaluate changes in continuous outcomes such as knowledge and attitude scores, while McNemar’s tests were used for binary outcomes related to the use of reflective jackets and functional lighting systems.

The primary evaluation of the intervention effect was conducted using a difference-in-differences approach implemented through generalized estimating equation models to account for repeated measurements over time. Logistic GEE models with a logit link were fitted for binary outcomes, while linear GEE models were used for continuous outcomes. Each model included study group, time, and a group-by-time interaction term representing the intervention effect. Both unadjusted and adjusted models were fitted. Adjusted models controlled for age group, marital status, education level, and years of riding experience. Adjusted odds ratios or regression coefficients with 95% confidence intervals were reported, and statistical significance was assessed at p < 0.05. Knowledge, attitude and practice scores were treated as continuous variables in all analyses and were not categorized, in order to preserve statistical power and avoid information loss.

### Post-intervention access for the control group

After completion of endline data collection, riders in the control district were offered access to the health education messages using the same mobile phone–based delivery approaches for a period of two months to ensure fairness and ethical balance.

## Results

### Baseline socio-demographic characteristics of study Participants by study group

A total of 183 riders were enrolled in the intervention Health District (Limbe) at baseline and 149 were retained at endline. A total of 130 riders were enrolled at baseline in the controlled Health District (Tiko) and 100 were retained at the end of the intervention. A total of 249 commercial motorcycle riders were therefore retained at the end of the health education intervention study comprising 149 riders from Limbe Health District and 100 riders from Tiko Health District. The mean age of riders in the Limbe and Tiko Health Districts were 33.4 ± 7.3 years and 32.0 ± 7.8 years respectively. Most riders in both health districts were aged 31–40 years, accounting for 43.6% in Limbe and 39.0% in Tiko. Overall, the age distribution of riders was broadly similar in both health districts, with the majority being younger adults (Table 1).

### Assessment of loss to follow-up (attrition analysis) in the Control and Intervention Groups

An attrition analysis was conducted to assess whether riders lost to follow-up differed systematically from those retained at endline in the intervention group (Limbe Health District) and control group (Tiko Health District). Baseline characteristics of riders who were retained and those lost to follow-up were compared using chi-square tests.

There was a statistically significant difference in attrition by age group (p = 0.029), indicating that loss to follow-up varied across age categories. Specifically, participants within the age group > 50 were all lost to follow up. However, no statistically significant differences were observed between retained and lost participants with respect to marital status (p = 0.989), level of education (p = 0.392) or years of riding (p = 0.663) (Table 2).

Overall, apart from age group, baseline socio-demographic characteristics were comparable between riders retained at endline and those lost to follow-up in Limbe.

In the Tiko health district, there was a statistically significant association between age group and attrition (p < 0.001), indicating that loss to follow-up differed across age categories. However, no statistically significant differences were observed between retained and lost participants with respect to marital status (p = 0.225), level of education (p = 0.980), average monthly income (p = 0.720), or years of riding (p = 0.148). Apart from age group, baseline characteristics were comparable between riders retained at endline and those lost to follow-up in Tiko.

### Within-group comparisons between baseline and endline

#### Within-group comparison of KAP scores in the control group (Tiko Health District)

Paired samples t-tests were conducted to compare baseline and endline scores for knowledge, attitudes and practices related to visibility materials among riders in the control group-Tiko Health District (Table 4). There were no statistically significant differences between baseline and endline scores for knowledge (mean difference = 0.07, t = 0.385, p = 0.701), attitudes (mean difference = 0.17, t = 0.430, p = 0.668), or practices (mean difference = − 0.13, t = 0.647, p = 0.519).

Overall, these findings indicate that knowledge, attitudes and practices related to visibility materials, did not change significantly.

#### Within-group comparison of visibility materials use at baseline and endline among riders in Tiko Health District

McNemar’s test was used to assess changes in the use of visibility materials between baseline and endline among commercial motorcycle riders in the control group-Tiko Health District (Table 5). There was no statistically significant change in the use of any of the visibility materials assessed between baseline and endline. Overall, these findings indicate that the use of visibility materials among riders in the control group did not change significantly over time.

#### Within-group comparison of KAP scores in the intervention group (Limbe Health District)

There were statistically significant increases in knowledge scores (mean difference = 1.75, t = 8.93, p < 0.001), attitude scores (mean difference = 5.60, t = 10.67, p < 0.001), and practice scores (mean difference = 0.36, t = 2.55, p = 0.012). Overall, these findings indicate that knowledge, attitudes and practices related to both visibility materials improved significantly over time among riders in the intervention group.

#### Within-group comparison of visibility materials use in the intervention group (Limbe Health District)

McNemar’s test was used to assess changes in the use of visibility materials between baseline and endline among commercial motorcycle riders in the intervention group-Limbe Health District (Table 7). There were statistically significant increases in the use of reflective jackets (p < 0.001), backlights (p = 0.004), brake lights (p = 0.023), and trafficators/indicator lights (p = 0.004) between baseline and endline. A borderline statistically significant increase in lamp use was also observed (p = 0.050). In contrast, there was no statistically significant change in the use of reflective strips between baseline and endline (p = 1.000). Overall, these findings indicate that the health education intervention was associated with significant improvements in the use of most visibility materials among riders in the intervention group, with the exception of reflective strips, for which no significant change was observed.

#### Difference-in-differences analysis of the impact of the health education intervention

To estimate the causal effect of the health education intervention on the use of visibility materials, a difference-in-differences (DiD) analytical approach was employed. The DiD method compares changes in outcomes over time in the intervention group (Limbe Health District) with changes over the same period in a comparable control group (Tiko Health District). This approach allows the effect of the intervention to be isolated from background temporal changes and other external influences that may affect both groups simultaneously, such as seasonal variations, enforcement activities or general public awareness activities.

#### Unadjusted difference-in-differences analysis of visibility materials use

Unadjusted difference-in-differences analyses using generalized estimating equations were conducted to assess the effect of the health education intervention on the use of visibility materials (Table 8). The intervention was associated with a statistically significant increase in reflective jacket use (COR = 4.21, 95% CI: 1.84–9.64, p = 0.001). No statistically significant intervention effects were observed for reflective strips use, headlamp use, backlight use, brake light use, or trafficator (indicator) light use (all p > 0.05).

The unadjusted models suggest that the health education intervention significantly improved reflective jacket use among commercial motorcycle riders, while no significant effects were observed for the other PPE and visibility materials.

#### Adjusted difference-in-differences analysis of PPE and visibility materials use

After adjusting for age group, level of education, average monthly income and riding experience, the health education intervention was associated with a statistically significant increase reflective jacket use (AOR = 4.34, 95% CI: 1.88–10.02, p = 0.001). No statistically significant intervention effects were observed for reflective strips use, headlamp use, backlight use, brake light use or trafficator (indicator) light use (all p > 0.05).

Overall, the adjusted models indicate that the health education intervention significantly improved reflective jacket use among commercial motorcycle riders, while no significant effects were observed for the other visibility materials assessed (Table 9).

### Difference-in-Differences Analysis for Continuous Outcomes

#### Unadjusted difference-in-differences analysis of KAP scores

Unadjusted difference-in-differences analyses using generalized estimating equations were conducted to assess the effect of the health education intervention on knowledge, attitudes and practices related to personal protective equipment and visibility materials. The intervention was associated with statistically significant increases in PPE knowledge scores (β = 2.91, 95% CI: 2.14–3.68, p < 0.001) and PPE attitude scores (β = 5.76, 95% CI: 4.32–7.21, p < 0.001). No statistically significant intervention effect was observed for PPE practice scores (β = 0.21, 95% CI: −0.09–0.52, p = 0.171). Similarly, statistically significant increases were observed in visibility materials knowledge scores (β = 1.68, 95% CI: 1.16–2.20, p < 0.001) and visibility materials attitude scores (β = 5.51, 95% CI: 4.22–6.80, p < 0.001). However, no statistically significant intervention effect was found for visibility materials practice scores (β = 0.34, 95% CI: −0.11 −0.79, p = 0.141). Overall, the unadjusted models indicate that the health education intervention significantly improved knowledge and attitudes related to both PPE and visibility materials, whereas improvements in practices were not statistically significant.

#### Adjusted difference-in-differences analysis of KAP scores

After adjusting for age group, marital status, level of education and years of riding, the health education intervention was associated with statistically significant improvements in knowledge and attitudes related to both personal protective equipment (PPE) and visibility materials (Table 10).

Specifically, PPE knowledge scores increased significantly in the intervention group compared with the control group (β = 2.91, 95% CI: 2.14–3.68, p < 0.001), as did PPE attitude scores (β = 5.76, 95% CI: 4.32–7.21, p < 0.001). However, no statistically significant intervention effect was observed for PPE practice scores (β = 0.21, 95% CI: −0.09–0.52, p = 0.171). Similarly, visibility materials knowledge (β = 1.68, 95% CI: 1.16–2.20, p < 0.001) and visibility materials attitude scores (β = 5.51, 95% CI: 4.22–6.80, p < 0.001) improved significantly following the intervention, whereas no statistically significant change was observed in visibility materials practice scores (β = 0.34, 95% CI: −0.11 −0.79, p = 0.141). These findings indicate that the health education intervention significantly improved riders’ knowledge and attitudes toward PPE and visibility materials but did not produce statistically significant changes in reported practices.

The similarity between the unadjusted and adjusted difference-in-differences estimates indicates that the measured socio-demographic variables did not confound the relationship between the health education intervention and the outcomes. The intervention effect therefore appears to be robust to adjustment for age, marital status, education and years of riding.

## Discussion

This quasi-experimental study evaluated the effectiveness of a theory-driven health education intervention in improving knowledge, attitudes, and use of visibility materials among commercial motorcycle riders in two health districts in Cameroon. Guided by the Health Belief Model and systematically developed using the Intervention Mapping approach, the intervention demonstrated a clear positive effect on riders’ knowledge and attitudes toward visibility materials and produced a statistically significant increase in reflective jacket use. In contrast, no meaningful changes were observed in the control district, indicating that the observed improvements were attributable to the intervention rather than to background temporal trends or external influences. Although attrition was observed, baseline characteristics were largely comparable between retained and lost participants, suggesting limited attrition bias.

The significant improvement in knowledge and attitudes related to visibility materials among riders in the intervention district is consistent with the theoretical assumptions of the Health Belief Model[15]. The intervention content explicitly targeted riders’ perceived susceptibility to road traffic injuries, perceived severity of crash outcomes, perceived benefits of visibility materials, and perceived barriers such as cost, discomfort and inconvenience. By repeatedly emphasizing the role of reflective jackets and functional lighting systems in reducing collision risk, particularly under low-visibility conditions, the intervention appears to have successfully modified riders’ cognitive and affective perceptions of visibility materials. This finding supports previous evidence that educational strategies grounded in behavioural theory can effectively influence safety-related beliefs among vulnerable road users.

The Intervention Mapping framework played an important role in translating these behavioural determinants into context-appropriate delivery strategies. The formative qualitative assessment conducted prior to implementation identified low risk perception, misconceptions regarding the usefulness of reflective materials and weak social norms around conspicuity as key barriers to adoption. These insights informed the development of locally relevant messages and delivery channels, including face-to-face sensitization and mobile phone–based reminders, which functioned as repeated cues to action. The significant gains observed in both knowledge and attitudes suggest that the participatory and structured planning process advocated by Intervention Mapping strengthened the relevance and acceptability of the intervention within the rider community.

Despite the substantial improvements in knowledge and attitudes, the adjusted difference-in-differences analysis showed that reflective jacket use was the only visibility material that improved significantly as a direct result of the intervention. While within-group analyses indicated increased use of backlights, brake lights and indicator lights among riders in the intervention district, these changes did not remain statistically significant after adjustment for secular trends and covariates. This pattern highlights an important distinction between short-term cognitive change and behavioural adoption, a gap that has been widely reported in injury prevention and road safety interventions[17].

From an HBM perspective, the limited translation of improved knowledge and attitudes into consistent behavioural change for most visibility materials suggests that perceived barriers may have continued to outweigh perceived benefits for some equipment. Reflective jackets represent a portable and relatively low-cost visibility measure that can be adopted without modifying the motorcycle itself[18]. In contrast, functional lighting systems and reflective strips require technical maintenance, replacement of damaged components or additional financial investment, which may have reduced riders’ perceived feasibility and self-efficacy for adopting these behaviours. These findings align with the self-efficacy construct of the Health Belief Model, which emphasises individuals’ confidence in their ability to perform a behaviour under existing constraints[19, 20].

The strong and statistically significant increase in reflective jacket use observed in the adjusted models further supports this interpretation. Reflective jackets were consistently emphasized during the intervention as a simple and immediately actionable safety practice. The repeated mobile phone reminders and demonstrations during group sessions likely served as effective cues to action, reinforcing daily behavioural intentions. This result is particularly important in the study context, where low lighting, congested roads and mixed traffic conditions are common and contribute substantially to crash risk among motorcycle riders[21–24].

The absence of a statistically significant intervention effect for reflective strips and motorcycle lighting components may also reflect structural and environmental limitations beyond individual control. Weak enforcement of road safety regulations, limited access to affordable spare parts and informal motorcycle repair practices were identified during the formative phase as important contextual barriers[25]. Intervention Mapping explicitly recognizes that behaviour change is influenced not only by individual determinants but also by environmental and organizational constraints. The present findings therefore underscore the need for complementary structural interventions, such as partnerships with local transport authorities, motorcycle unions and repair workshops, to improve availability and affordability of visibility equipment.

Importantly, no significant changes in visibility materials knowledge, attitudes or use were observed in the control district. This strengthens causal inference and supports the internal validity of the study. The lack of spill-over effects further suggests that the measures implemented to minimize contamination between districts were effective.

Although visibility materials practice scores did not demonstrate a statistically significant intervention effect in the adjusted models, the observed improvements in reflective jacket use represent an important and meaningful behavioural outcome. In road safety research, even modest increases in conspicuity have been associated with reductions in collision risk, particularly in low-light and high-traffic environments[26, 27]. The present findings therefore have practical relevance for injury prevention programming in similar urban and peri-urban settings in low- and middle-income countries.

This study contributes to the limited body of experimental evidence on conspicuity-focused interventions among commercial motorcycle riders in sub-Saharan Africa. Most existing intervention studies in the region have concentrated primarily on helmet use and general road safety practices, with limited attention to visibility materials despite their importance for crash prevention. By explicitly integrating visibility materials into both theoretical framing and intervention content, this study expands the scope of behavioural road safety interventions.

Several limitations should be considered when interpreting the findings. First, the quasi-experimental design, although strengthened by the difference-in-differences analytical approach, does not provide the same level of causal control as a randomized trial. Second, visibility materials use were assessed through direct observation, some practice measures relied on self-report and may be subject to social desirability bias. Third, the follow-up period captured short-term behavioural change, and it remains unclear whether observed improvements, particularly in reflective jacket use, will be sustained over time. Finally, the study was conducted in two districts within a single region, which may limit generalizability to other settings with different road infrastructure and regulatory environments.

Nevertheless, the study demonstrates that a health education intervention systematically developed through Intervention Mapping and grounded in the Health Belief Model can significantly improve visibility-related knowledge, attitudes, and selected safety behaviours among commercial motorcycle riders. Future interventions should integrate educational strategies with structural and policy-level measures aimed at improving access to affordable visibility equipment, strengthening enforcement and engaging motorcycle unions as partners in sustaining safety norms. Such multilevel approaches are likely to be necessary to translate improved awareness and motivation into broader and more sustained adoption of visibility materials for injury prevention.

## Conclusion

This study shows that a health education intervention developed through the Intervention Mapping approach and guided by the Health Belief Model significantly improved commercial motorcycle riders’ knowledge and attitudes toward visibility materials in Limbe Health District. The intervention also produced a significant increase in reflective jacket use, indicating improved conspicuity behaviour among riders. However, no significant intervention effects were observed for most motorcycle lighting and reflective components, including reflective strips and functional lighting systems. These findings indicate that theory-based educational interventions can enhance awareness and motivation to use visibility materials but may be insufficient on their own to overcome structural and resource-related constraints affecting the adoption of certain visibility devices. Integrating educational strategies with broader regulatory, enforcement and access-oriented measures is likely necessary to achieve sustained improvements in rider visibility and injury prevention. These findings are relevant to other low- and middle-income settings where commercial motorcycle transport is common and road safety systems are underdeveloped.

## Supplementary Material

This is a list of supplementary files associated with this preprint. Click to download.

• SupplementaryInformation.docx

• CLEANDIDDATASET.sav

## Figures and Tables

**Table 1:** Socio-demographic characteristics of retained riders in the control and intervention groups

Variable		Health District		Total
	Category	Limbe	Tiko	
Age group (years)	41–50	25(16.8)	8(8)	33(13.3)
	31–40	65(43.6)	39(39)	104(41.8)
	21–30	58(38.9)	50(50)	108(43.4)
**Total**	**Total**	**148(99.3)**	**97(97)**	**245(98.4)**
Marital status	Married	80(53.7)	38(38)	118(47.4)
	Single	69(45.3)	62(62)	126(50.6)
**Total**	**Total**	**149(100)**	**100(100)**	**249(100)**
Highest education	Tertiary	14(9.4)	5(5)	19(7.6)
	Secondary	81(54.4)	61(61)	142(57)
	Primary	43(28.9)	30(30)	73(29.3)
	No formal	11(7.4)	4(4)	15(6)
**Total**	**Total**	**149(100)**	**100(100)**	**249(100)**
Average monthly income (XAF1000)	> 150	11(7.4)	8(8)	19(7.6)
	101–150	47(31.5)	27(27)	74(29.7)
	50–100	85(57)	61(61)	146(58.6)
	< 50	6(4)	4(4)	10(4)
**Total**	**Total**	**149(100)**	**100(100)**	**249(100)**
Riding duration (years)	> 10	26(17.4)	17(17)	43(17.3)
	5–10	48(32.2)	29(29)	77(30.9)
	< 5	75(50.3)	54(54)	129(51.8)
	**Total**	**149(100)**	**100(100)**	**249(100)**

**Table 2:** Baseline characteristics of riders retained and lost to follow-up in Limbe Health District

Variable	Category	Lost to follow-up n (%)	Retained at endline n (%)	p-value
Age group (years)	**> 50**	**3(100.0)**	**0(0.0)**	**0.029**
	41–50	0 (0.0)	25 (100.0)	
	31–40	16 (19.8)	65 (80.2)	
	21–30	**18 (23.7)**	58 (76.3)	
Marital status	Married	18 (18.4)	80 (81.6)	0.989
	Single	16 (19.8)	69 (80.2)	
Education level	Tertiary	4 (22.2)	14 (77.8)	0.392
	Secondary	21 (20.6)	81 (79.4)	
	Primary	9 (17.3)	43 (82.7)	
	No formal	0 (0.0)	11 (100.0)	
Years of riding	> 10	4 (13.3)	26 (86.7)	0.663
	5–10	12 (20.0)	48 (80.0)	
	< 5	18 (19.4)	75 (80.6)	

* Percentages are calculated within each category

**Table 3:** Baseline characteristics of riders retained and lost to follow-up in Tiko Health District

Variable	Category	Lost to follow-up n (%)	Retained at endline n (%)	p-value
Age group (years)	> 50	5 (100.0)	0(0.0)	**< 0.001**
	41–50	10 (55.6)	8 (44.4)	
	31–40	14 (25.9)	40 (74.1)	
	21–30	4 (7.5)	49 (92.5)	
Marital status	Married	14 (26.4)	39 (73.6)	
	Single	16 (20.0)	61 (80.0)	
Highest education level	Tertiary	2 (28.6)	5 (71.4)	0.98
	Secondary	16 (21.1)	60 (78.9)	
	Primary	11 (26.2)	31 (73.8)	
	No formal	1 (20.0)	4 (80.0)	
Average monthly income (in 1000XAF)	> 150	3 (27.3)	8 (72.7)	0.72
	101–150	11 (28.9)	27 (71.1)	
	50–100	15 (19.7)	61 (80.3)	
	< 50	1 (20.0)	4 (80.0)	
Years of riding (years)	> 10	9 (36.0)	16 (64.0)	0.148
	5–10	10 (25.0)	30 (75.0)	
	< 5	11 (16.9)	54 (83.1)	

* Percentages are calculated within each category/ row

**Table 4:** Within-group comparison of KAP scores in the control group (Tiko Health District)

Outcome	Mean difference (Endline-Baseline)	t	p-value
Visibility material knowledge	0.07	0.385	0.701
Visibility material attitude	0.17	0.43	0.668
Visibility material practice	0.13	0.647	0.519

* Paired t-test; mean difference represents endline minus baseline scores.

**Table 5:** Within-group comparison of visibility materials use at baseline and endline among riders in Tiko Health District (n = 100)

Visibility materials	Baseline Use (%)	Endline Use (%)	% Change (Endline-Baseline)	p-value
Backlight	72.0	79.6	7.6	0.883
Jacket	11.0	15.5	4.5	0.324
Strips	84.0	88.8	4.8	0.424
Lamp	47.0	49.5	2.5	0.523
Brake light	55.0	53.5	−1.5	0.883
Trafficator	35.0	32.3	−2.7	0.775

* p-values were obtained using McNemar's test. Percentages (%) are based on n = 100 riders

**Table 6:** Within-group comparison of KAP scores in the intervention group (Limbe Health District)

Outcome	Mean difference (Baseline - Endline)	t	p-value
Visibility material knowledge	1.75	8.93	**< 0.001**
Visibility material attitude	5.6	10.67	**< 0.001**
Visibility material practice	0.36	2.55	**0.012**

* Paired t-test; mean difference represents endline minus baseline scores.

**Table 7:** Within-group comparison of visibility materials use at baseline and endline among riders in Limbe Health District (n = 149)

Visibility material	Baseline use (%)	Endline Use (%)	(%) Change (Endline-Baseline)	p-value
Reflective jacket	72.0	79.6	7.6	< 0.001
Reflective strips	11.0	15.5	0.7	1.000
Lamp	84.0	88.8	8.1	0.050
Backlight	47.0	49.5	6.0	**0.004**
Brake light	47.0	53.5	6.5	**0.023**
Trafficator (indicator)	35.0	32.3	6.0	**0.004**

**Table 8:** Unadjusted difference-in-differences (GEE) estimates of the effect of the intervention on PPE and visibility materials use

Outcome		95% CI for COR		
	COR (Group × Time)	Lower	Upper	p value
Jacket use	4.210	1.838	9.641	**0.001**
Strips use	0.756	0.240	2.378	0.632
Headlamp use	1.408	0.505	3.930	0.513
Backlight use	1.146	0.653	2.010	0.635
Brake light use	0.620	0.312	1.231	**0.172**
Four trafficator (indicator) lights use	1.415	0.748	2.676	0.286

* COR = crude odds ratio from GEE logistic difference-in-differences models. The intervention effect is represented by the Group × Time interaction term

**Table 9:** Adjusted difference-in-differences analysis of PPE and visibility materials use

Outcome	AOR (Group × Time)	95% CI	p-value
Reflective jacket use	4.34	1.88–10.02	**0.001**
Reflective strips use	0.75	0.23–2.44	0.634
Headlamp use	1.35	0.56–3.24	0.501
Backlight use	1.15	0.65–2.06	0.632
Brake light use	0.61	0.30–1.24	0.173
Trafficator (indicator) light use	1.45	0.74–2.83	0.282

* AOR = adjusted odds ratio from GEE logistic difference-in-differences models. Models were adjusted for age group, level of education, average monthly income and riding experience. The intervention effect is represented by the Group × Time interaction term

**Table 10:** Unadjusted difference-in-differences (GEE) estimates of the effect of the intervention on KAP scores

Outcome	β (Group × Time)	95% CI	p-value
Visibility materials knowledge score	1.68	1.16–2.20	**< 0.001**
Visibility materials attitude score	5.51	4.22–6.80	**< 0.001**
Visibility materials practice score	0.34	−0.11–0.79	0.141

* All models adjusted for age group, marital status, education and years of riding.

* **β** *represents the difference-in-differences estimate from linear GEE models. The intervention effect is represented by the Group × Time interaction term*.
